# Associations Among Educational Attainment, Health Literacy, and Diet Quality Among Korean Adults

**DOI:** 10.3390/nu18213643

**Published:** 2026-11-01

**Authors:** Seong-Uk Baek, Jin-Ha Yoon

**Affiliations:** 1 Graduate School, Yonsei University College of Medicine, Seoul 03722, Republic of Korea; 2 Department of Preventive Medicine, Yonsei University College of Medicine, Seoul 03722, Republic of Korea; 3 Institute for Innovation in Digital Healthcare, Yonsei University Health System, Seoul 03722, Republic of Korea; 4 The Institute for Occupational Health, Yonsei University College of Medicine, Seoul 03722, Republic of Korea

**Keywords:** educational attainment, health literacy, diet quality, diet pattern

## Abstract

**Background:** Accumulating evidence has demonstrated disparities in dietary quality according to educational attainment. This study examined associations among educational attainment, health literacy (HL), and dietary quality. **Methods:** This cross-sectional study included a nationally representative sample of 9943 adults. Educational attainment was categorized into four groups: elementary school or lower, middle school, high school, and college or higher. HL was assessed using the Health Literacy Index for the Community (HLIC) scale, and dietary quality was assessed using the Korean Healthy Eating Index (KHEI). Linear regression models were used to determine the association between educational attainment and KHEI. Mediation analysis was conducted to examine the indirect association of educational attainment with KHEI score through the HLIC, expressed as indirect association. Beta (β) coefficients and 95% confidence intervals (CIs) were computed. **Results:** Of the participants, 15.2% had completed elementary school or lower, 10.1% middle school, 33.2% high school, and 41.5% had attained college education or higher. While an inverse association was observed between educational attainment and KHEI score, the direction of the association reversed to positive after adjustment for age. In the fully adjusted model, using elementary school or lower as the reference group, middle school, high school, and college or higher education were associated with 2.46-point (95% CI, 1.30–3.61), 2.43-point (95% CI, 1.44–3.42), and 2.86-point (95% CI, 1.81–3.92) higher KHEI scores, respectively. The adjusted β coefficients (95% CI) for the indirect association were β = 0.19 (95% CI, 0.10–0.30) for middle school, β = 0.40 (95% CI, 0.24–0.57) for high school, and β = 0.53 (95% CI, 0.31–0.76) for college or higher, accounting for 7.70%, 16.61%, and 18.48% of the total association, respectively. **Conclusions:** This study showed an indirect association between educational attainment and dietary quality via HL. However, longitudinal studies should be conducted to elucidate causal mediation.

## 1. Introduction

Health literacy (HL) refers to an ability to access and apply health knowledge to promote health, considering personal circumstances and the broader healthcare context [1]. HL can be developed through various channels, including formal education and school curricula, communication with healthcare providers, and digital and community resources [2,3,4,5]. Previous studies have found that HL is inversely associated with healthcare costs [6], cardiovascular disease risk [7], and mortality risk [8]. HL also plays an important role in shaping favorable health behaviors. For instance, higher HL has been positively associated with physical activity and participation in health checkups and negatively associated with cigarette smoking [9,10,11]. 

Improving dietary quality is important for preventing and managing health conditions, such as cardiovascular diseases and metabolic disorders. Dietary quality can be assessed using indices that capture multiple dimensions of dietary intake while incorporating culturally relevant and local dietary guidelines [12]. A recent study, for example, reported a decline in dietary quality in the Korean population, as measured by the Korean Healthy Eating Index (KHEI) [13]. This trend highlights the importance of effective public health policies to improve dietary quality, particularly among vulnerable populations, including older adults and individuals with low socioeconomic status. 

**Figure 1:**
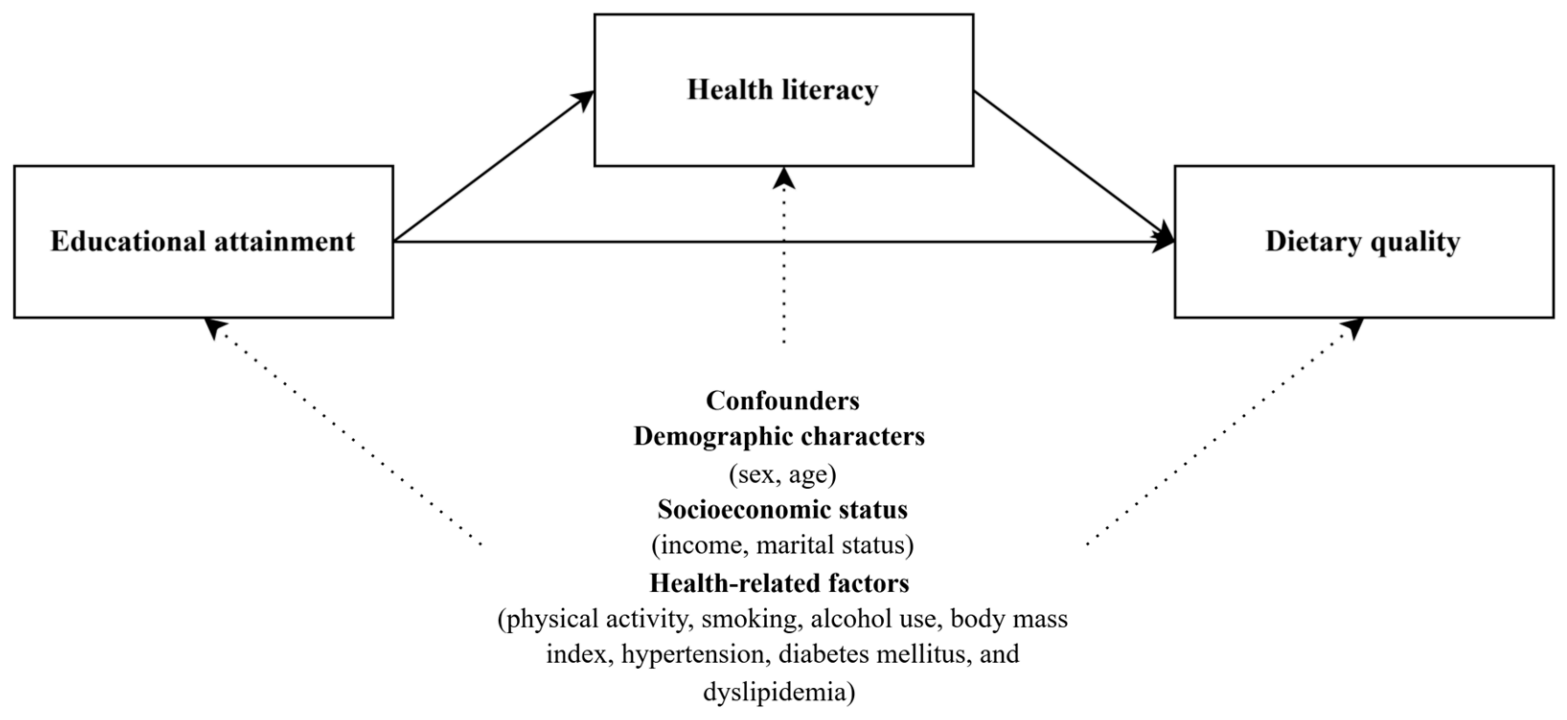
Conceptual framework of the potential relationship among educational attainment, health literacy, and dietary quality in this study.

Among the social determinants of dietary quality, educational attainment has been identified as an important structural factor associated with various health conditions and dietary quality [14,15,16,17]. The mechanisms underlying educational disparities in dietary quality may be complex. One potential pathway is knowledge-based: higher educational attainment may facilitate a better understanding of the health implications of different foods and greater access to healthier food options [17,18]. Therefore, HL may account for the association between educational attainment and dietary quality (Figure 1).

However, few studies have examined the potential role of HL in this association. A study of Danish adults found that HL partially mediated the association between educational attainment and a healthy diet [19]. Similarly, a study of Belgian adults found that HL significantly mediated this association [20]. However, these studies focused on European populations [19,20], and evidence from South Korea and other Asian populations remains limited. Given the cultural and regional variation in educational systems and dietary practices, the associations among educational attainment, HL, and dietary quality may differ across settings. In our previous study, we demonstrated the role of HL in associations of educational attainment with aerobic physical activity and muscle-strengthening exercise [21]. In contrast, the present study extends this work by examining whether HL also serves as a potential pathway linking educational attainment to dietary quality, a distinct health behavior assessed using the KHEI. A recent study in South Korea revealed a positive association between HL and diet quality measured by the KHEI after adjustment for age and other socio-demographic characteristics [22]. However, an indirect association between educational attainment, a key indicator of socioeconomic position, and dietary quality through HL has not been examined in a Korean context. Therefore, examining these associations in a Korean setting may provide novel insights into how these relationships vary across cultural contexts, beyond population-specific replication. Furthermore, it remains unclear how an indirect association via HL varies across levels of educational attainment. This study aimed to examine associations among educational attainment, HL, and dietary quality among adults in South Korea.

## 2. Methods

### 2.1. Study Sample

**Figure 2:**
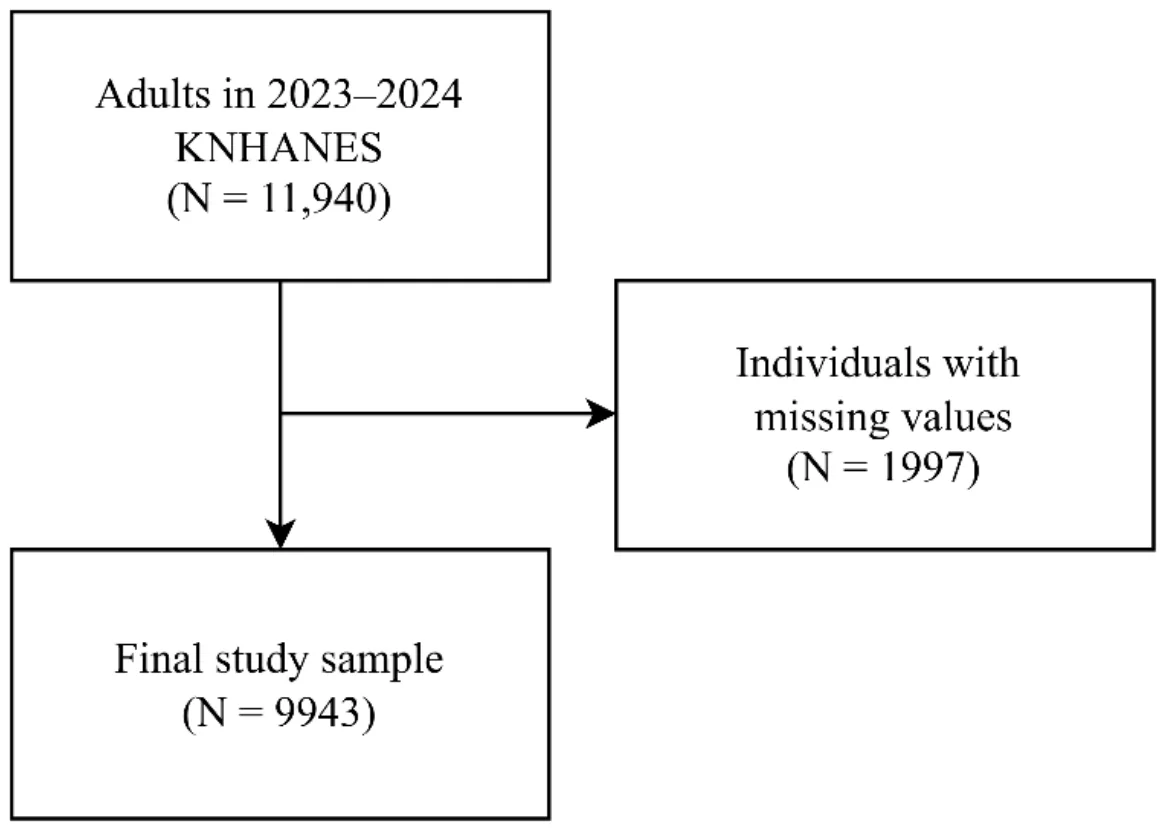
Flow diagram of the sample selection.

The present study employed datasets from the Korea National Health and Nutrition Examination Survey (KNHANES), a nationwide cross-sectional survey representative of the Korean population. Participants from the 2023 and 2024 cycles of the KNHANES were included, during which survey items related to HL were collected. The KNHANES employs a multistage cluster sampling design to recruit individuals from South Korea [23]. A flow diagram illustrating the sample selection process is shown in Figure 2. Initially, 11,940 individuals aged >18 years participated in the KNHANES nutritional survey. After excluding 1997 individuals with missing data, 9943 adults were included in this study. Written informed consent was obtained from all participants. The Institutional Review Board of the Korea Disease Control and Prevention Agency granted ethical approval (2022–11–16–R–A; 2022–11–16–R–03).

### 2.2. Main Variables

Based on the Korean 6-3-3-4 single-ladder educational system, the participants’ educational attainment was self-reported and classified as elementary school or lower (reference category), middle school, high school, and college or higher [24].

HL was measured using the Health Literacy Index for the Community (HLIC) [25]. The HLIC includes 10 items assessing self-reported abilities to understand and use health information related to disease prevention, health promotion, healthcare utilization, and the use of technology and health resources. Responses to the 10 items are scored on a 4-point Likert scale, resulting in a total score of 10–40, with higher scores representing greater HL. A prior study has demonstrated good reliability and validity of the HLIC in the Korean population [25]. Cronbach’s alpha was 0.92 in the sample, suggesting good reliability. The mean HLIC score was 30.6 (standard deviation, 4.9), with scores ranging from 10 to 40. Participants with a missing HLIC score were excluded from the primary analysis, and the missing value was addressed using multiple imputation in the sensitivity analysis.

This study used the KHEI, a validated measure for assessing dietary quality in the Korean population [26]. The KHEI consists of 14 items that assess dietary quality across three domains: adequacy, moderation, and energy balance. The adequacy component includes eight items assessing the intake of mixed grains, total fruits, fresh fruits, total vegetables, vegetables excluding kimchi and pickled vegetables, meat, fish, eggs, beans, dairy products, and breakfast. The moderation component includes three items assessing the moderate intake of saturated fat, sodium, and sugars. Finally, the energy balance component includes three items related to the percentage of energy from fat, percentage of energy from carbohydrates, and total daily energy intake. The KHEI score ranges from 0 to 100, where higher scores represent greater dietary quality. Nutritional assessments were performed using 24-h dietary recall, with trained dietitians assisting participants to improve recall accuracy. Details of the specific KHEI items and their scoring system have been described previously [26], and are also provided in Table S1.

### 2.3. Control Variables

The following variables were included as covariates in the analysis: sex, age (continuous scale), income (Q1, lowest, to Q4, highest), marital status (married, unmarried, and others [divorced, separated, or widowed]), physical activity (<150 min/wk, ≥150 min/wk), smoking status (no, yes), alcohol use (Alcohol Use Disorders Identification Test-Consumption, AUDIT-C), body mass index (BMI, kg/m^2^), hypertension (no, yes), diabetes mellitus (no, yes), dyslipidemia (no, yes), and survey year (2023, 2024).

Specifically, income levels were grouped using quartiles of average personal monthly income for each survey year, using the variable “incm”. Physical activity was measured based on the Global Physical Activity Questionnaire (Korean version) [27]. BMI was calculated using objectively measured height and weight. Hypertension was defined as current use of antihypertensive medication, systolic blood pressure ≥ 140 mmHg, or diastolic blood pressure ≥ 90 mmHg. Diabetes was defined as the current use of oral hypoglycemic agents, insulin, or a hemoglobin A1C level ≥ 6.5%. Dyslipidemia was defined as the use of lipid-lowering medications or a total cholesterol level ≥ 240 mg/dL. Measurements and categorizations were consistent across survey cycles.

Covariates were selected based on their theoretical relevance to the relationships among educational attainment, HL, and dietary quality, supported by previous literature [17]. Additionally, variables previously considered as covariates in studies examining education, HL, and health behaviors were included [19,22]. However, some variables, particularly health status, may lie on the proposed causal pathway between educational attainment and dietary quality. Therefore, to assess the potential influence of confounding and the robustness of the findings, models were sequentially fitted: a model adjusted for sex and age, a model additionally adjusted for socioeconomic status (income and marital status), and a fully adjusted model including all selected covariates. The results from all three models are presented.

### 2.4. Data Analysis

#### 2.4.1. Preliminary Analysis

Data analysis was performed using R software (version 4.6.0). For the preliminary analysis, linear regression models were employed to examine the association between educational attainment and KHEI score. Four linear regression models were sequentially fitted: a crude model, a sex-adjusted model, a sex- and age-adjusted model, and a fully adjusted model that included all prespecified confounders.

Subsequently, two associations constituting the indirect pathway, namely, the associations between educational attainment and HLIC score and between HLIC and KHEI scores, were examined using linear regression models, adjusting for confounders. The associations were presented as beta coefficients (βs) and 95% confidence intervals (CIs). Measurement and categorizations for all variables were consistent across the survey cycles.

#### 2.4.2. Mediation Analysis

The complex survey design was specified in the R “survey” package [28]. Specifically, sampling weights (variable name: “wt_tot”), strata (“kstarta”), and primary sampling units (“psu”) were specified in the “svydesign” function. When pooling the two survey cycles, sampling weights were divided by two to account for the number of survey cycles combined. Mediation analysis was conducted to analyze the potential role of HL in the association between educational attainment and dietary quality. The counterfactual framework-based mediation analysis was performed using the R package “mediation” [29]. This procedure decomposed the total association into an indirect association through HL and a direct association not accounted for by HL. Additionally, the proportion mediated (PM) was calculated, and 95% confidence intervals (CIs) were computed. In the first step, two separate models were fitted using “svyglm”: a mediator model regressing HL on educational attainment and covariates, and an outcome model regressing dietary quality on educational attainment, HL, and covariates. Subsequently, quasi-Bayesian Monte Carlo simulation with 5000 iterations was used to estimate counterfactual outcomes based on simulated coefficients and their variance–covariance matrices obtained from the survey-weighted regression models fitted in the preceding step. Three separate mediation analyses were conducted, each comparing one of the educational categories (middle school, high school, college or higher) with the reference category (elementary or lower). In each educational category, the indirect, direct, and total associations were estimated. Model 1 was adjusted for sex and age. Model 2 was additionally adjusted for income and marital status. Model 3 was further adjusted for physical activity, smoking, alcohol use, body mass index, hypertension, diabetes, dyslipidemia, and survey year. For the sensitivity analysis, missing data were handled using multiple imputation to generate 20 complete datasets using the R package “mice” [30]. Survey-weighted regression models were fitted using “svyglm” for each imputed dataset, followed by quasi-Bayesian Monte Carlo simulation with 1000 iterations to estimate the indirect and direct associations and their corresponding variances. The estimates and variance estimates across the imputed datasets were pooled using Rubin’s rules implemented in the “MIcombine” function of the “mitools” package. Additionally, a sensitivity analysis was conducted using a restricted cubic spline function with five knots for age to account for potential nonlinear relationships. Given the marked differences in age distribution across educational attainment groups, analyses were further stratified by median age (≤55 vs. >55 years). A trend analysis treating educational attainment as an ordinal score was conducted as a supplementary analysis. The mediation analyses were repeated using the raw data without survey weights as a sensitivity analysis. Finally, mediation analysis allowing for an exposure–mediator interaction was performed.

## 3. Results

Sample characteristics across educational attainment categories are shown in Table 1. Compared with individuals with an elementary school education or lower, those with higher educational attainment tended to be men, younger, have higher income levels, and engage in physical activity, but were less likely to have chronic conditions. The mean KHEI scores were 64.0, 63.8, 57.2, and 57.1 among participants with elementary school or lower, middle school, high school, and college or higher education, respectively. In contrast, the mean HLIC scores across the corresponding educational attainment groups were 26.6, 28.3, 30.6, and 31.8, respectively. Table S2 compares the characteristics of the included and excluded samples. Compared with included participants, excluded participants were more likely to be older, have lower educational attainment and income levels, and have a lower mean HLIC score. 

Table 2 presents the association of educational attainment with KHEI scores. In the unadjusted model (Model 1), with elementary school or lower as the reference category, the β coefficients (95% CIs) for the KHEI were −0.19 (−1.53, 1.15), −6.81 (−7.79, −5.84), and −6.91 (−7.88, −5.95) for middle school, high school, and college or higher, respectively. The direction of the associations reversed after age was included in the model (Model 3), with β coefficients (95% CIs) of 2.51 (1.33, 3.70), 2.96 (1.98, 3.95), and 4.09 (3.05, 5.13), respectively. This suggests that age acts as an important confounder in the association between educational attainment and KHEI score. In the fully adjusted model (Model 4), the corresponding β coefficients (95% CIs) were 2.46 (1.30, 3.61), 2.43 (1.44, 3.42), and 2.86 (1.81, 3.92), respectively.

Table 3 presents the associations of educational attainment with HL and of HL with dietary quality. Using elementary school or lower as the reference category, β coefficients (95% CIs) for the HLIC were 1.44 (0.98–1.89) for middle school, 3.02 (2.64–3.40) for high school, and 3.94 (3.55–4.33) for college or higher (Model 1). In Model 2, HLIC score was positively associated with KHEI score (β = 0.16; 95% CI, 0.10–0.21).

Table 4 presents the results of the main analysis. For the indirect association between educational attainment and dietary quality, the β coefficient (95% CIs) was 0.19 (0.10–0.30) for middle school, 0.40 (0.24–0.57) for high school, and 0.53 (0.31–0.76) for college or higher, using elementary school or lower as the reference group. The corresponding PMs (95% CIs) were 7.70% (3.77–16.05%), 16.61% (8.88–31.86%), and 18.48% (10.00–32.86%), respectively.

Results from the analysis using multiple imputations are shown in Table S3. The estimates for the indirect association and PM were greater in the models using multiple imputation. For example, in Model 3, the β value (95% CI) for the indirect association was 0.39 (0.29–0.49) for middle school, 0.65 (0.42–0.89) for high school, and 0.80 (0.52–1.09) for college or higher, using elementary school or lower as the reference group. The corresponding PMs (95% CIs) were 13.47% (5.53–21.40%), 23.18% (8.95–31.59%), and 25.64% (12.95–38.34%), respectively. The sensitivity analysis using a restricted cubic spline function for age yielded results similar to those observed in the main analysis (Table S4). In the age-stratified analyses (Table S5), the β values (95% CIs) for the indirect associations were generally lower in the younger age group (<55 years), at 0.11 (−0.12–0.41) for middle school, 0.29 (0.04–0.66) for high school, and 0.37 (0.09–0.77) for college or higher. In the older age group (≥55 years), the corresponding estimates were 0.26 (0.14–0.41), 0.51 (0.30–0.73), and 0.81 (0.50–1.13), respectively. The trend analysis treating educational attainment as an ordinal score (Table S6) yielded an indirect association of 0.17 (95% CI: 0.09–0.24) and a PM of 22.06% (95% CI: 11.19–41.52%) in the fully adjusted model. In the sensitivity analysis not using survey weights, the indirect association was slightly attenuated and the direct association increased; the positive association for the indirect association was maintained (Table S7). Finally, in the sensitivity analysis allowing for an exposure–mediator interaction, positive indirect associations were observed across the middle school, high school, and college or higher groups (Table S8).

## 4. Discussion

Using a nationally representative sample of Korean adults, this study found an indirect association between educational attainment and dietary quality via HL. However, longitudinal studies are needed to clarify the temporal relationships among educational attainment, HL, and dietary quality.

The findings of our study are consistent with previous evidence of a positive association between HL and dietary quality [31,32,33,34]. A systematic review found that higher HL was consistently associated with healthier dietary choices and better dietary quality [35]. A recent study of South Korean adults reported an inverse association between HL and KHEI score before adjustment for age, whereas the direction of the association became positive after age adjustment [22]. Our findings support this observation, highlighting the importance of age as a potential confounder in the association between HL and KHEI score. Additionally, our findings are consistent with the theoretical framework suggesting a potential indirect association of socioeconomic position with health disparities through HL [36]. In our previous work, HLIC score partially accounted for educational disparities in adherence to WHO-recommended physical activity guidelines [21]. The present study extends this work by suggesting that the potential mediating role of the HLIC may extend beyond physical activity to other health behaviors, including dietary quality. A study of Danish adults found that HL partially mediated the association between educational attainment and lifestyle behaviors, including smoking, physical inactivity, unhealthy dietary habits, and obesity [19]. Similarly, a study of Belgian adults found that HL partially mediated the association between educational attainment and lifestyle behaviors, including physical activity, healthy diet, alcohol use, and tobacco use [20]. The PMs for diet were 13.8% and 3.8% in the Danish and Belgian studies, respectively [19,20], which were relatively lower than those observed in the present study among participants with middle school and college or higher education. Although these previous studies provide meaningful contributions to the literature, they did not examine how the indirect associations varied across different categories of educational attainment [19,20]. Therefore, the present study extends previous research by demonstrating similar indirect associations in a nationally representative sample of Korean adults and by examining how these associations varied across educational attainment categories.

Multiple mechanisms may underpin the associations among educational attainment, HL, and dietary quality. Formal education may enhance critical thinking and facilitate the development of health-related skills, thereby increasing individuals’ capacity to acquire and use health-related knowledge and potentially increasing HL [37,38]. While the HLIC evaluates general HL rather than HL specific to food or nutrition, general HL may be associated with favorable dietary quality through several competencies. According to the theoretical framework described in previous studies, various domains of HL may be associated with favorable food choices and dietary quality [39]. Functional HL encompasses basic knowledge of health and nutrition, which may facilitate understanding of dietary recommendations. Interactive HL encompasses the cognitive skills required to seek and apply dietary information in daily decision-making. Critical HL involves the ability to critically evaluate dietary information and exercise greater control over dietary decisions [39]. Therefore, individuals with high HL may be associated with better dietary quality. However, the present analysis assessed indirect association through the HLIC total score, which reflects overall HL. Therefore, the findings should not be interpreted as evidence that a specific HL mechanism accounts for the observed associations. In addition to HL-related factors, alternative socioeconomic pathways may also be relevant to the association between educational attainment and dietary quality. For instance, in a Korean context, educational attainment may be associated with broader social networks and greater opportunities for social support [40]. Therefore, individuals with higher educational attainment may have greater access to social networks and support, which may be associated with better dietary quality [41]. Additionally, in South Korea, educational attainment plays an important role in shaping occupational and social positions [40,42]. In contrast, lower educational attainment may be associated with greater exposure to unfavorable working conditions, such as irregular hours, shift work, and long working hours, which may contribute to poorer dietary quality [43]. However, these potential mechanisms remain hypothetical and should be formally examined in future studies.

While the present study showed an indirect association between educational attainment and dietary quality via HL, the absolute effect sizes were modest, with estimates smaller than 1 point on the 100-point KHEI scale. Additionally, most of the adjusted associations were independent of health literacy, indicating direct associations. Although no established threshold exists for a meaningful difference in KHEI score, the observed differences were modest relative to the standard deviation (SD =13.2), suggesting that statistical significance does not necessarily imply clinical significance, although these differences may still be relevant at the population level.

The present study had limitations. First, causal relationships between educational attainment, HL, and dietary quality could not be claimed owing to the cross-sectional study design. The mediation analysis relied on several assumptions, including sequential ignorability and the absence of unmeasured confounding in the relationships among exposure, mediator, and outcome. Temporal ordering among exposure, mediator, and outcome is required to estimate true mediating effects. Therefore, the findings should be interpreted as exploratory rather than evidence of causal mediation. Second, although the KHEI is a validated tool of dietary quality, it is based on a 24-h dietary recall and is therefore susceptible to recall bias and may not adequately capture long-term dietary quality or day-to-day variations in dietary practices. A single 24-h dietary recall may not adequately capture usual dietary intake, as dietary patterns often vary by weekday versus weekend and by season. These variations were not accounted for in the present study. Although trained dietitians guided participants to improve the accuracy of dietary recall and assessment, future studies should employ more objective measures to better capture long-term dietary patterns and habits. Third, causal mediation analysis assumes no unmeasured confounding factors in the relationships among exposure, mediator, and outcome. However, educational attainment may reflect broader socioeconomic circumstances, including neighborhood conditions, occupational environments, social capital, and parental socioeconomic position, which have been associated with dietary quality [44,45]; however, these factors could not be considered in this study because such information was unavailable. Fourth, because HLIC scores were assessed using self-reported functional literacy, all items may be susceptible to differential measurement error by educational level. For example, individuals with higher educational attainment may rate their functional literacy more favorably. Such systematic errors may bias the estimated indirect association.

Despite these limitations, this study also had several strengths. First, we analyzed a nationally representative sample, which may have enhanced robustness and generalizability. Second, the present study is one of few to examine the potential role of HL in associations of educational attainment with dietary quality among Korean adults, which is an understudied population in the existing literature.

## 5. Conclusions

This study investigated associations among educational attainment, HL, and dietary quality. Notably, higher educational attainment is indirectly associated with dietary quality through pathways involving HL. Therefore, these findings highlight the importance of HL in explaining disparities in dietary quality across educational attainment levels.

## Figures and Tables

**Table 1:** Sample characteristics across educational attainment.

	Overall	Educational Attainment			
		Elementary School or Lower	Middle School	High School	College or Higher
	*n* = 9943	*n* = 1508	*n* = 1003	*n* = 3302	*n* = 4130
Sex					
Male	4290 (50.0%)	467 (34.0%)	419 (45.9%)	1483 (51.5%)	1921 (53.3%)
Female	5653 (50.0%)	1041 (66.0%)	584 (54.1%)	1819 (48.5%)	2209 (46.7%)
Age (years)					
Mean (SD)	49.0 (16.8)	71.1 (7.6)	64.6 (10.6)	46.4 (17.2)	43.3 (12.8)
Income level					
Lowest	2348 (23.4%)	472 (30.3%)	306 (30.5%)	814 (25.9%)	756 (18.8%)
Low	2518 (25.3%)	422 (27.1%)	274 (26.5%)	881 (27.6%)	941 (23.1%)
High	2514 (25.3%)	357 (24.4%)	217 (21.9%)	848 (24.5%)	1092 (26.7%)
Highest	2563 (26.0%)	257 (18.2%)	206 (21.1%)	759 (22.0%)	1341 (31.4%)
Marital status					
Married	6603 (62.7%)	911 (61.4%)	758 (75.3%)	2095 (57.0%)	2839 (65.0%)
Unmarried	1950 (26.4%)	22 (1.9%)	38 (5.1%)	809 (32.9%)	1081 (30.7%)
Others	1390 (10.9%)	575 (36.7%)	207 (19.6%)	398 (10.1%)	210 (4.3%)
Physical activity					
<150 min/week	5429 (51.3%)	1107 (71.7%)	664 (65.9%)	1749 (50.1%)	1909 (45.0%)
≥150 min/week	4514 (48.7%)	401 (28.3%)	339 (34.1%)	1553 (49.9%)	2221 (55.0%)
Smoking					
No	8521 (83.4%)	1390 (91.3%)	851 (81.9%)	2693 (79.1%)	3587 (84.9%)
Yes	1422 (16.6%)	118 (8.7%)	152 (18.1%)	609 (20.9%)	543 (15.1%)
AUDIT-C					
Mean (SD)	3.8 (3.5)	1.9 (3.1)	3.0 (3.5)	4.1 (3.6)	4.1 (3.4)
Body mass index (kg/m^2^)					
Mean (SD)	24.2 (3.9)	24.5 (3.3)	24.4 (3.6)	24.2 (4.0)	24.0 (4.0)
Hypertension					
No	6625 (71.5%)	558 (37.6%)	444 (45.9%)	2276 (73.0%)	3347 (82.5%)
Yes	3318 (28.5%)	950 (62.4%)	559 (54.1%)	1026 (27.0%)	783 (17.5%)
Diabetes mellitus					
No	8400 (86.8%)	1095 (72.3%)	731 (74.2%)	2810 (87.1%)	3764 (91.9%)
Yes	1543 (13.2%)	413 (27.7%)	272 (25.8%)	492 (12.9%)	366 (8.1%)
Dyslipidemia					
No	6745 (71.8%)	800 (52.6%)	539 (55.4%)	2256 (72.7%)	3150 (78.2%)
Yes	3198 (28.2%)	708 (47.4%)	464 (44.6%)	1046 (27.3%)	980 (21.8%)
KHEI					
Mean (SD)	58.4 (13.2)	64.0 (12.1)	63.8 (13.0)	57.2 (13.5)	57.1 (12.7)
HLIC					
Mean (SD)	30.6 (4.9)	26.6 (5.6)	28.3 (4.5)	30.6 (4.5)	31.8 (4.5)

SD, standard deviation; AUDIT-C, Alcohol Use Disorders Identification Test-Consumption; KHEI, Korean Healthy Eating Index; HLIC, Health Literacy Index for the Community. Values were presented as unweighted n with survey-weighted % for categorical variables and survey-weighted mean with standard deviation.

**Table 2:** Association between educational attainment and Korean Healthy Eating Index score using linear regression models.

	Model 1	Model 2	Model 3	Model 4
	β (95% CI)	β (95% CI)	β (95% CI)	β (95% CI)
**Educational attainment**				
Elementary school or lower	Reference	Reference	Reference	Reference
Middle school	−0.19 (−1.53, 1.15)	−0.01 (−1.35, 1.32)	2.51 (1.33, 3.70)	2.46 (1.30, 3.61)
High school	−6.81 (−7.79, −5.84)	−6.55 (−7.52, −5.58)	2.96 (1.98, 3.95)	2.43 (1.44, 3.42)
College or higher	−6.91 (−7.88, −5.95)	−6.62 (−7.59, −5.66)	4.09 (3.05, 5.13)	2.86 (1.81, 3.92)
p-for-trend	< 0.001	< 0.001	< 0.001	< 0.001

CI, confidence interval; Model 1, unadjusted model; Model 2, adjusted for sex; Model 3, adjusted for sex and age; Model 4, adjusted for sex, age, income, marital status, smoking, physical activity, alcohol use, body mass index, hypertension, diabetes mellitus, dyslipidemia, and survey year.

**Table 3:** Associations of educational attainment with health literacy and of health literacy with dietary quality.

Independent Variable	Dependent Variable	β	95% CI
**Model 1 (Pathway: Education → Health literacy)**			
Elementary school or lower	HLIC	Reference	Reference
Middle school		1.44	0.98, 1.89
High school		3.02	2.64, 3.40
College or higher		3.94	3.55, 4.33
**Model 2 (Pathway: Health literacy → Dietary quality)**			
HLIC	KHEI	0.16	0.10, 0.21

HLIC, Health Literacy Index for the Community; KHEI, Korean Healthy Eating Index; CI, confidence interval. All models are adjusted for sex, age, income, marital status, physical activity, smoking, alcohol use, body mass index, hypertension, diabetes, dyslipidemia, and survey year.

**Table 4:** Total, indirect, and direct associations among educational attainment, health literacy, and dietary quality.

	Total Association	Indirect Association	Direct Association	Proportion Mediated
	β (95% CI)	β (95% CI)	β (95% CI)	% (95% CI)
**Model 1**				
**Educational attainment**				
Elementary school or lower	Reference	Reference	Reference	Reference
Middle school	2.52 (1.36, 3.67)	0.28 (0.17, 0.41)	2.24 (1.06, 3.42)	10.89 (5.87, 22.67)
High school	2.96 (1.98, 3.93)	0.58 (0.38, 0.78)	2.39 (1.39, 3.36)	19.57 (11.77, 31.99)
College or higher	4.08 (3.07, 5.13)	0.78 (0.52, 1.04)	3.31 (2.26, 4.39)	19.10 (12.00, 28.40)
**Model 2**				
**Educational attainment**				
Elementary school or lower	Reference	Reference	Reference	Reference
Middle school	2.21 (1.01, 3.36)	0.23 (0.13, 0.35)	1.97 (0.81, 3.16)	10.34 (5.12, 24.07)
High school	2.45 (1.48, 3.41)	0.49 (0.30, 0.68)	1.97 (0.95, 2.94)	19.90 (11.31, 35.76)
College or higher	3.29 (2.25, 4.35)	0.65 (0.42, 0.89)	2.63 (1.59, 3.72)	19.87 (11.70, 32.31)
**Model 3**				
**Educational attainment**				
Elementary school or lower	Reference	Reference	Reference	Reference
Middle school	2.46 (1.32, 3.58)	0.19 (0.10, 0.30)	2.28 (1.12, 3.42)	7.70 (3.77, 16.05)
High school	2.42 (1.44, 3.41)	0.40 (0.24, 0.57)	2.02 (1.01, 3.03)	16.61 (8.88, 31.86)
College or higher	2.86 (1.85, 3.92)	0.53 (0.31, 0.76)	2.34 (1.26, 3.40)	18.48 (10.00, 32.86)

CI, confidence interval. Model 1 was adjusted for sex and age. Model 2 was adjusted for sex, age, income and marital status. Model 3 was adjusted for sex, age, income, marital status physical activity, smoking, alcohol use, body mass index, hypertension, diabetes, dyslipidemia, and survey year.

## Data Availability

Data can be found at https://knhanes.kdca.go.kr (accessed on 3 August 2026).

## Supplementary Materials

The following supporting information can be downloaded at https://www.mdpi.com/article/10.3390/nu18213643/s1, Table S1: Scoring system and characteristics of the Korean Healthy Eating Index in the study sample; Table S2: Comparison of the sample characteristics between included and excluded samples; Table S3: Total, indirect, and direct associations among educational attainment, health literacy, and dietary quality using multiple imputations; Table S4: Total, indirect, and direct associations among educational attainment, health literacy, and dietary quality using a restricted cubic spline function for age; Table S5: Total, indirect, and direct associations among educational attainment, health literacy, and dietary quality. Stratified analysis by age; Table S6: The results of the trend analysis treating educational attainment as an ordinal score; Table S7: Sensitivity analysis using models using the unweighted raw data; Table S8: Sensitivity analysis using the mediation model allowing for exposure–mediator interaction. File S1. Supplementary details. R codes for mediation analysis.
