# Supplementary material for: Associations Among Educational Attainment, Health Literacy, and Diet Quality Among Korean Adults
Source: Nutrients. 2026 Nov 1;18(21):3643. doi: 10.3390/nu18213643 (PMC13765102; doi:10.3390/nu18213643)
Supplement: Supplementary file 1 [file nutrients-18-03643-s001.zip › nutrients-4623312-supplementary.pdf]

Table S1. Scoring system and characteristics of the Korean Healthy Eating Index in the study sample

	Scoring		
	Score	Criteria	
		Minimum score	Maximum score
**Total KHEI score**	0 to 100	0	100
**Adequacy component**			
**Total adequacy score**			
1.Have breakfast	0 to 10	0 times/wk	≥5 times/wk
2.Mixed grains	0 to 5	0 serving/d	≥0.3 serving/d
3.Total fruits (men)	0 to 5	0 serving/d	≥3/2 (<65 yrs/≥65 yrs) serving/d
3.Total fruits (women)	0 to 5	0 serving/d	≥2/1 (<65 yrs/≥65 yrs) serving/d
4.Fresh fruits (men)	0 to 5	0 serving/d	≥1.5/1 (<65 yrs/≥65 yrs) serving/d
4.Fresh fruits (women)	0 to 5	0 serving/d	≥1/0.5 (<65 yrs/≥65 yrs) serving/d
5.Total vegetables (men)	0 to 5	0 serving/d	≥8 serving/d
5.Total vegetables (women)	0 to 5	0 serving/d	≥8/6 (<65 yrs/≥65 yrs) serving/d
6.Vegetable intake excluding kimchi and pickled vegetables (men)	0 to 5	0 serving/d	≥5 serving/d
6.Vegetable intake excluding kimchi and pickled vegetables (women)	0 to 5	0 serving/d	≥5/3 (<65 yrs/≥65 yrs) serving/d
7.Meat, fish, eggs, and beans (men)	0 to 10	0 serving/d	≥5/4 (<65 yrs/≥65 yrs) serving/d
7.Meat, fish, eggs, and beans (women)	0 to 10	0 serving/d	≥4/2.5 (<65 yrs/≥65 yrs) serving/d
8.Milk and milk products intake	0 to 10	0 serving/d	≥1 serving/d
**Moderation component**			
**Total moderation score**			
9.Percentage of energy from saturated fatty acids	0 to 10	>10% of total energy intake	<7% of total energy intake
10.Sodium	0 to 10	>6,500 mg/d	≤2,000 mg/d
11.Percentage of energy from sugars	0 to 10	>20% of total energy intake	≤10% of total energy intake
**Energy balance component**			
**Total energy balance score**			
12.Percentage of energy intake from carbohydrate	0 to 5	<50% or >75% of total energy intake	55–65% of total energy intake
13.Percentage of energy intake from fat	0 to 5	<10% or >35% of total energy intake	15–30% of total energy intake
14.Energy intake	0 to 5	<60% or >140% of the EER	75–125% of the EER

KHEI, Korean Healthy Eating Index; EER, estimated energy requirement.

Table S2 Comparison of the sample characteristics between included and excluded samples

	Included sample	Excluded sample	*p*-value
	n = 9,943	n = 1,997	
Sex			0.876
Male	4,290 (43.1%)	866 (43.4%)	
Female	5,653 (56.9%)	1,131 (56.6%)	
Age (years)			<0.001
Mean (SD)	53.2 (16.7)	58.3 (17.3)	
Educational attainment			<0.001
Elementary school or lower	1,508 (15.2%)	397 (24.5%)	
Middle school	1,003 (10.1%)	177 (10.9%)	
High school	3,302 (33.2%)	523 (32.3%)	
College or higher	4,130 (41.5%)	522 (32.2%)	
Missingness	0	378	
Income level			<0.001
Lowest	2,348 (23.6%)	623 (32.3%)	
Low	2,518 (25.3%)	446 (23.1%)	
High	2,514 (25.3%)	454 (23.5%)	
Highest	2,563 (25.8%)	408 (21.1%)	
Missingness	0	66	
Marital status			<0.001
Married	6,603 (66.4%)	1,237 (62.7%)	
Unmarried	1,950 (19.6%)	268 (13.6%)	
Others	1,390 (14.0%)	469 (23.8%)	
Missingness	0	23	
Physical activity			<0.001
<150 min/week	5,429 (54.6%)	569 (61.3%)	
≥150 min/week	4,514 (45.4%)	359 (38.7%)	
Missingness	0	1,069	
Smoking			<0.001
No	8,521 (85.7%)	1,345 (82.3%)	
Yes	1,422 (14.3%)	290 (17.7%)	
Missingness	0	362	
AUDIT-C			0.010
Mean (SD)	3.4 (3.5)	3.1 (3.6)	
Missingness	0	354	
Body mass index (kg/m^2)			0.679
Mean (SD)	24.1 (3.8)	24.0 (3.9)	
Missingness	0	217	
Hypertension			<0.001
No	6,625 (66.6%)	1,019 (56.0%)	
Yes	3,318 (33.4%)	800 (44.0%)	
Missingness	0	178	
Diabetes mellitus			<0.001
No	8,400 (84.5%)	901 (76.6%)	
Yes	1,543 (15.5%)	276 (23.4%)	
Missingness	0	820	
Dyslipidemia			0.255
No	6,745 (67.8%)	808 (66.2%)	
Yes	3,198 (32.2%)	413 (33.8%)	
Missingness	0	776	
KHEI			0.570
Mean (SD)	60.0 (13.1)	60.2 (12.5)	
Missingness	0	303	
HLIC			<0.001
Mean (SD)	30.3 (5.1)	25.1 (9.0)	
Missingness	0	3	

P values were calculated using chi-squared test for categorical variables and -t-test for continuous variables.

Table S3 Total, indirect, and direct associations among educational attainment, health literacy, and dietary quality using multiple imputations

	Indirect association	Direct association	Proportion mediated
	**β (95% CI)**	**β (95% CI)**	**% (95% CI)**
**Model 1**			
**Educational attainment**			
Elementary school or lower	Reference	Reference	Reference
Middle school	0.57 (0.38, 0.75)	2.57 (1.50, 3.64)	18.07 (8.99, 27.15)
High school	0.95 (0.69, 1.21)	2.53 (1.63, 3.43)	27.24 (16.7, 37.71)
College or higher	1.21 (0.88, 1.54)	3.35 (2.36, 4.34)	26.45 (17.31, 35.60)
**Model 2**			
**Educational attainment**			
Elementary school or lower	Reference	Reference	Reference
Middle school	0.48 (0.31, 0.65)	2.22 (1.14, 3.30)	17.84 (7.09, 28.60)
High school	0.81 (0.56, 1.06)	2.05 (1.13, 2.97)	28.45 (15.36, 41.55)
College or higher	1.02 (0.72, 1.32)	2.61 (1.61, 3.61)	28.09 (16.55, 39.63)
**Model 3**			
**Educational attainment**			
Elementary school or lower	Reference	Reference	Reference
Middle school	0.39 (0.29, 0.49)	2.51 (2.21, 2.82)	13.47 (5.53, 21.40)
High school	0.65 (0.42, 0.89)	2.16 (1.89, 2.43)	23.18 (8.95, 31.59)
College or higher	0.80 (0.52, 1.09)	2.33 (2.02, 2.64)	25.64 (12.95, 38.34)

CI, confidence interval;

Model 1 was adjusted for sex and age. Model 2 was adjusted for sex, age, income and marital status. Model 3 was adjusted for sex, age, income, marital status physical activity, smoking, alcohol use, body mass index, hypertension, diabetes, dyslipidemia, and survey year.

Table S4 Total, indirect, and direct associations among educational attainment, health literacy, and dietary quality using a restricted cubic spline function for age

	Indirect association	Direct association	Proportion mediated
	β (95% CI)	β (95% CI)	% (95% CI)
**Model 1**			
**Educational attainment**			
Elementary school or lower	Reference	Reference	Reference
Middle school	0.24 (0.13, 0.36)	1.71 (0.50, 2.92)	12.10 (5.96, 33.94)
High school	0.54 (0.35, 0.73)	2.41 (1.39, 3.47)	18.27 (10.46, 29.99)
College or higher	0.74 (0.48, 1.00)	4.03 (2.83, 5.22)	15.46 (9.69, 23.32)
**Model 2**			
**Educational attainment**			
Elementary school or lower	Reference	Reference	Reference
Middle school	0.21 (0.11, 0.32)	1.48 (0.25, 2.60)	12.07 (5.36, 38.91)
High school	0.47 (0.28, 0.66)	1.92 (0.83, 2.95)	19.35 (10.58, 39.59)
College or higher	0.63 (0.40, 0.88)	3.26 (2.17, 4.41)	16.09 (9.42, 24.89)
**Model 3**			
**Educational attainment**			
Elementary school or lower	Reference	Reference	Reference
Middle school	0.17 (0.09, 0.27)	1.57 (0.37, 2.76)	9.86 (4.28, 27.83)
High school	0.39 (0.22, 0.56)	1.59 (0.61, 2.69)	19.73 (9.43, 43.27)
College or higher	0.51 (0.28, 0.74)	2.39 (1.27, 3.54)	17.80 (9.17, 31.98)

CI, confidence interval

Model 1 was adjusted for sex and age. Model 2 was adjusted for sex, age, income and marital status. Model 3 was adjusted for sex, age, income, marital status physical activity, smoking, alcohol use, body mass index, hypertension, diabetes, dyslipidemia, and survey year.

Table S5 Total, indirect, and direct associations among educational attainment, health literacy, and dietary quality. Stratified analysis by age

	**Indirect association**	**Direct association**	**Proportion mediated**
	**β (95% CI)**	**β (95% CI)**	**% (95% CI)**
**Age ≤ 55 years**			
**Educational attainment**			
Elementary school or lower	Reference	Reference	Reference
Middle school	0.11 (-0.12, 0.41)	-1.80 (-7.09, 3.86)	-1.92 (-59.17, 85.76)
High school	0.29 (0.04, 0.66)	0.90 (-2.85, 4.84)	9.82 (-167.74, 153.45)
College or higher	0.37 (0.09, 0.77)	1.56 (-2.17, 5.60)	12.55 (-164.81, 181.90)
**Age > 55 years**			
**Educational attainment**			
Elementary school or lower	Reference	Reference	Reference
Middle school	0.26 (0.14, 0.41)	2.61 (1.16, 3.46)	10.27 (5.29, 20.09)
High school	0.51 (0.30, 0.73)	2.03 (0.89, 3.09)	20.00 (11.00, 37.70)
College or higher	0.81 (0.50, 1.13)	1.67 (0.33, 2.94)	32.87 (17.06, 72.10)

CI, confidence interval;

All models were adjusted for sex, age, income, marital status physical activity, smoking, alcohol use, body mass index, hypertension, diabetes, dyslipidemia, and survey year.

Table S6 The results of the trend analysis treating educational attainment as an ordinal score.

	**Indirect association**	**Direct association**	**Proportion mediated**
	**β (95% CI)**	**β (95% CI)**	**% (95% CI)**
**Model 1**			
**Educational attainment**			
Trend	0.25 (0.17, 0.34)	0.97 (0.62, 1.33)	20.33 (12.72, 30.87)
**Model 2**			
**Educational attainment**			
Trend	0.21 (0.13, 0.29)	0.76 (0.41, 1.09)	21.59 (12.33, 36.64)
**Model 3**			
**Educational attainment**			
Trend	0.17 (0.09, 0.24)	0.60 (0.26, 0.96)	22.06 (11.19, 41.52)

CI, confidence interval;

Model 1 was adjusted for sex and age. Model 2 was adjusted for sex, age, income and marital status. Model 3 was adjusted for sex, age, income, marital status physical activity, smoking, alcohol use, body mass index, hypertension, diabetes, dyslipidemia, and survey year.

Table S7 Sensitivity analysis using models using the unweighted raw data

	Indirect association	Direct association	Proportion mediated
	β (95% CI)	β (95% CI)	% (95% CI)
**Model 1**			
**Educational attainment**			
Elementary school or lower	Reference	Reference	Reference
Middle school	0.24 (0.15, 0.35)	2.87 (1.91, 3.79)	7.73 (4.46, 12.97)
High school	0.51 (0.33, 0.68)	3.10 (2.25, 3.89)	13.93 (8.61, 20.90)
College or higher	0.69 (0.48, 0.92)	3.96 (3.15, 4.78)	14.93 (10.25, 20.86)
**Model 2**			
**Educational attainment**			
Elementary school or lower	Reference	Reference	Reference
Middle school	0.19 (0.12, 0.29)	2.52 (1.57, 3.49)	7.10 (3.98, 12.97)
High school	0.40 (0.26, 0.56)	2.54 (1.75, 3.35)	13.76 (8.07, 21.43)
College or higher	0.54 (0.35, 0.74)	3.17 (2.33, 4.09)	14.66 (8.97, 21.68)
**Model 3**			
**Educational attainment**			
Elementary school or lower	Reference	Reference	Reference
Middle school	0.16 (0.08, 0.24)	2.67 (1.77, 3.59)	5.50 (2.77, 9.73)
High school	0.33 (0.17, 0.48)	2.47 (1.64, 3.27)	11.77 (6.15, 20.56)
College or higher	0.44 (0.23, 0.64)	2.70 (1.81, 3.60)	14.00 (7.39, 23.49)

CI, confidence interval;

Model 1 was adjusted for sex and age. Model 2 was adjusted for sex, age, income and marital status. Model 3 was adjusted for sex, age, income, marital status physical activity, smoking, alcohol use, body mass index, hypertension, diabetes, dyslipidemia, and survey year.

Table S8 Sensitivity analysis using the mediation model allowing for exposure-mediator interaction.

	Indirect association (average)	Direct association (average)	Proportion mediated (average)
	β (95% CI)	β (95% CI)	% (95% CI)
**Model 1**			
**Educational attainment**			
Elementary school or lower	Reference	Reference	Reference
Middle school	0.47 (0.26, 0.72)	1.96 (0.73, 3.16)	19.04 (9.47, 39.88)
High school	0.84 (0.56, 1.13)	2.02 (1.01, 3.02)	29.54 (17.67, 48.30)
College or higher	1.10 (0.74, 1.46)	2.87 (1.83, 3.94)	27.92 (17.19, 40.94)
**Model 2**			
**Educational attainment**			
Elementary school or lower	Reference	Reference	Reference
Middle school	0.38 (0.20, 0.61)	1.72 (0.53, 2.85)	17.83 (8.62, 42.54)
High school	0.71 (0.46, 0.97)	1.57 (0.55, 2.58)	31.16 (17.52, 58.85)
College or higher	0.90 (0.58, 1.24)	2.25 (1.10, 3.35)	28.58 (17.06, 49.02)
**Model 3**			
**Educational attainment**			
Elementary school or lower	Reference	Reference	Reference
Middle school	0.38 (0.20, 0.57)	1.96 (0.85, 3.10)	15.79 (7.80, 33.17)
High school	0.64 (0.39, 0.89)	1.62 (0.63, 2.61)	28.41 (15.25, 52.83)
College or higher	0.80 (0.51, 1.12)	1.90 (0.79, 2.93)	29.41 (17.31, 52.76)

CI, confidence interval;

Model 1 was adjusted for sex and age. Model 2 was adjusted for sex, age, income and marital status. Model 3 was adjusted for sex, age, income, marital status physical activity, smoking, alcohol use, body mass index, hypertension, diabetes, dyslipidemia, and survey year.

File S1. Supplementary details. R codes for mediation analysis

Fitting fully-adjusted model

model_med<-svyglm(HealthLiteracy ~ Education + sex + age + income + marriage + pa_aerobic + smoke + audit + HE_BMI + HTNc + DMc + DLDc + year , design=data, family=gaussian())

model_out<-svyglm(HEI ~ Education + HealthLiteracy + sex + age + income + marriage + pa_aerobic + smoke + audit + HE_BMI + HTNc + DMc + DLDc + year , design=data, family=gaussian())

med_result_a <- mediation::mediate(model_med, model_out, treat = " Education", control.value = "Elementary", treat.value = "MiddleSchool", mediator = "HealthLiteracy", sims = 5000)

med_result_b <- mediation::mediate(model_med, model_out, treat = "Education", control.value = "Elementary", treat.value = "HighSchool", mediator = "HealthLiteracy", sims = 5000)

med_result_c<- mediation::mediate(model_med, model_out, treat = "Education", control.value = "Elementary", treat.value = "CollegeorHigher", mediator = "HealthLiteracy", sims = 5000)

summary(med_result_a)

summary(med_result_b)

summary(med_result_c)
